# Development of healthcare use across contemporary retirement pathways: results from a register based cohort study

**DOI:** 10.1177/1403494821998901

**Published:** 2021-03-19

**Authors:** Stefanie König, Susanne Kelfve, Andreas Motel-Klingebiel, Martin Wetzel

**Affiliations:** 1 Department of Psychology and Centre for Ageing and Health – AgeCap, University of Gothenburg, Gothenburg, Sweden; 2 Division Ageing and Social Change, Department of Culture and Society, Linköping University, Linköping, Sweden; 3 Aging Research Center, Karolinska Institutet & Stockholm University, Stockholm, Sweden; 4 Institute of Sociology and Social Psychology, University of Cologne, Cologne, Germany

**Keywords:** Healthcare disparities, retirement, public health, Sweden, socio-economic factors, healthy ageing, cohort studies

## Abstract

**Aim:**

We aimed to understand the interplay between retirement pathways and healthcare use in the postponed and structurally changing context of retirement.

**Methods:**

Based on Swedish register data on income and healthcare use, we applied combined sequence and cluster analysis to identify typical pathways into retirement and analysed their relation to healthcare use developments.

**Results:**

We detected five distinct pathways into retirement. Level of healthcare use was significantly higher for the pathway via disability pensions. We saw an overall increase in healthcare use across the retirement process that was related to age rather than to the different pathways.

**Conclusions:**

Level of healthcare use at the beginning of the retirement process may be related to selection into different pathways of retirement. We did not find clear evidence across several healthcare measures that different pathways lead to different developments in healthcare use.

## Introduction

Many studies have investigated how health affects retirement [1] and how retirement affects health [2], however, study findings have been inconclusive. We argue that previous research has often lacked a detailed understanding of how retirement proceeds. Previous studies defined retirement as an event rather than as a variety of retirement pathways during which a transitional process of detaching from work takes place. This distinction is crucial because health developments and retirement processes may occur simultaneously. Unarguably, these processes affect each other, but less as a process based on causality than of mutual interference. Following life course research, it is important to understand how these life course domains travel together. This study used Swedish register data on income sources to identify typical pathways of retirement transitions, and also register data on healthcare to identify typical patterns of retirement-related health developments.

Research on how retirement pathways and health are interrelated can contribute to better understanding individuals’ wellbeing in later life, while also providing information for healthcare budgets. Understanding the variety of these interrelations has become even more important because Sweden’s pension system has undergone several reforms aimed at prolonging working lives by restricting early retirement pathways and incentivizing later exits, which will lead to a further diversification of retirement pathways. Health and healthcare use are strongly correlated [3]. This study used healthcare use as a proxy for health since it is a more comparable and objective measure of health status. In addition, investigating healthcare instead of health gave us the opportunity to use register data, which circumvents selection bias from dropouts due to poor health [4].

Our core research question was: Do people in different pathways of retirement show different patterns of healthcare use during this process?

### Previous research on health and retirement

The relationship between retirement and health is complex. While research agrees on health being a strong determinant for retirement [1], results on the effect of retirement on health are inconsistent [2]. Some studies provide evidence for a beneficial effect of retirement on health, others find no or even negative effects. While these disparities may be related to different conceptualizations, operationalizations and measurements, one major limitation of previous studies is the deficient understanding of retirement, which is not a singular shift but a more or less stretched status passage. Little is known about the relationship between the timing of the retirement passage and healthcare use. Existing studies focus on early pension withdrawal or disability pensions [5–7]. From a theoretical standpoint, different effects from the timing of retirement can be expected [8]. The causal relationship between health and retirement is challenging from a methodological perspective. For example, health changes before retirement might cause labour market exits, which will remain undetected in longitudinal studies with lengthier intervals. Furthermore, in a survey design, selection bias might influence the results since ill health could lead to selective attrition, in particular among older people [9].

To our knowledge, no previous study has tried to understand the relationship between retirement and health using a differentiated process-based definition of retirement. The argument that loss of work strain may be beneficial relies heavily on the notion that retirement happens as a one-time event and ignores the possibility of gradual transitions. Even if timing of retirement is considered, this strongly depends on whether retirement age is defined as first pension receipt or as having no income from paid work. We argue that this neglects gradual retirement transitions and can lead to blurred results.

### Current study

Defining when a person is retired has been long debated [10]. The definition of retirement is even more complex in countries with less standardized and statutorily regulated retirement transitions in which individuals differ widely in when and how they retire. Therefore, Sweden is an interesting case to study as it employs a flexible retirement age that allows state pension withdrawals from the age of 61 to 67. In today’s retirement cohorts, retirement transitions are more flexible than in previous cohorts and this may even increase in future cohorts [11, 12].

Research on retirement has focused more intensely on methods that offer a better understanding of these diverse pathways, such as a more conceptional approach [13] or statistical sequence analyses [14–16]. To our knowledge, simultaneously analysing health development and retirement pathways has not yet been undertaken. We used sequence pattern analysis to classify individual successions into clusters of similar retirement pathways [14, 17, 18]. Sequence analysis examines the similarity of patterns of discrete statuses by estimating how many changes in a current position or how many positional changes would be necessary to produce equal patterns. Based on the estimated distances between each sequence compared with all other sequences, clusters of similar patterns can be identified. To examine the interconnection of labour market status sequences with healthcare use development, the current study analysed cluster-specific health developments over time and between groups. We assumed that pathways would differ in the extent and type of healthcare use and that development would vary over the retirement process.

We focused on one particular birth cohort born in 1947 that had reached the age of 61 in 2008 and 68 in 2015. To address the gradual steps involved in retiring in Sweden in our retirement definition, we applied an income-based indicator of retirement, which is based on an individual’s income sources. Following Fasang’s definition of retirement trajectories as ‘the sequence of primary income sources within the age bracket during which old-age pension entrance is theoretically possible’ [17], we defined retirement as a transitional process instead of a singular step.

## Data and method

This study is based on Swedish register data. Information about labour market status, sources of income and socio-demographic factors was extracted from LISA, a longitudinal database from Statistics Sweden for the total Swedish population. Information about inpatient care (number of nights in hospital) and specialized outpatient care (number of visits to the doctor with diagnoses, including day surgery and psychiatric care) was extracted from the National Patient Register. This register is administrated by the Swedish National Board of Health and Welfare and is estimated to include more than 99% of all inpatient hospital discharges [19].

We followed the cohort born in 1947 between the ages of 60 and 68. As sequence pattern analysis is a complex procedure that requires extensive computing power, we selected a 5% random sample of all individuals born in 1947 who were living in Sweden between 1990 and 2015 (*n* = 7000). We deleted individuals with incomplete data due to migration (*n* = 487) or death (*n* = 414), as well as individuals where it was not possible to identify the main income source. The final sample consisted of 5900 individuals with full information for the study period.

Based on four potential income sources (gainful work, self-employment, old-age pension and disability pension), we defined seven discrete labour market statuses: (1) main income from self-employment, (2) main income from a salary, (3) more than 50% income from a salary or self-employment (while also receiving a pension), (4) more than 50% of income from a pension (while also having income from a salary or self-employment), (5) main income from a pension, (6) main income from a disability pension and (7) other sources or low income. To assign other cases of mixed statuses, we used the largest income source as the primary income (see also Jönsson et al. [20]). Minimal income was based on the price base amount (*prisbasbelopp*), which is calculated according to the consumer price index and used for legal regulations on, for example, guarantee pensions.

In order to capture the complexity of the succession of seven different labour market statuses over 8 years, we applied sequence pattern analysis to identify typical pathways of transition. We used Stata 15 with the SQ-ados [21]. We applied the conventional optimal matching algorithm as it has been shown to be unbiased for classifications focusing on the timing of different transitions [22]. We used the transition-based probability matrix for identifying substitution costs and defined indel costs of 1. More details on development of the sequence analysis model and its identification strategy can be found in Supplemental material. We opted for a five-class solution.

## Results

**Figure 1.**
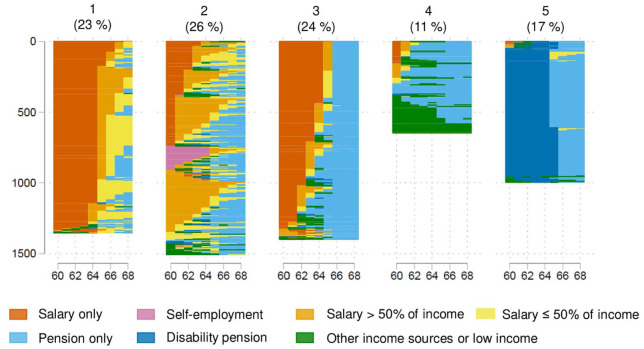
Retirement pathways in five clusters.

The sequence analysis resulted in five clusters, describing distinct pathways into retirement as shown in Figure 1.

### Description of sequences

**Figure 2.**
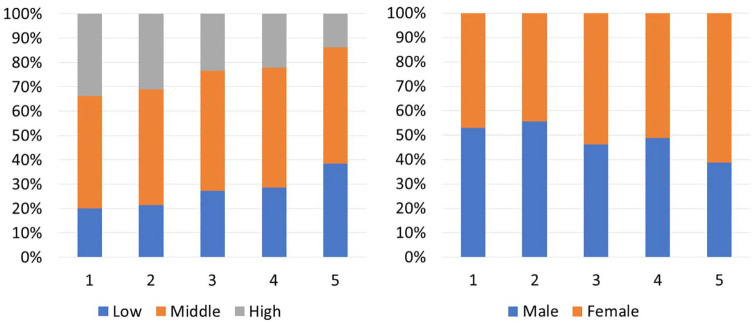
Distribution of educational level (left) and gender (right) by cluster.

**Figure 3.**
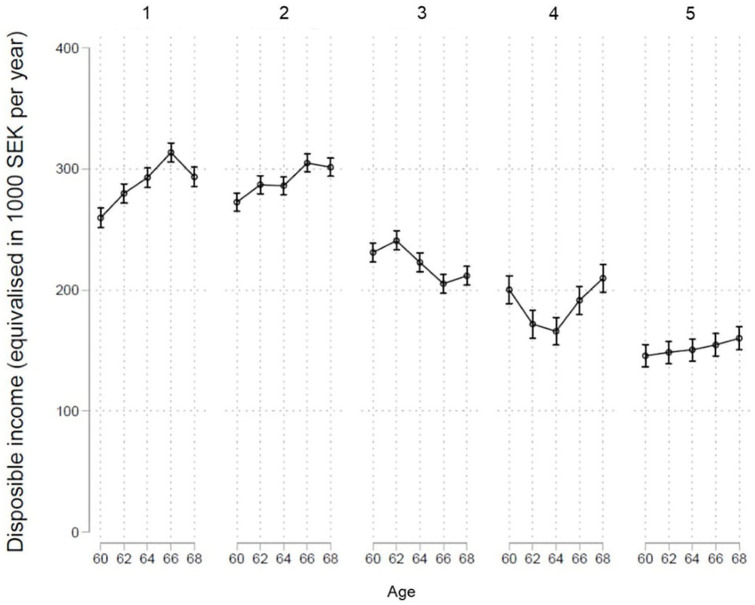
Income trajectories by cluster.

Figures 2 and 3 provide an overview of the constellation of social groups and income trajectories within each cluster. This is important background information since some studies show differences for healthcare use across social groups that is related to access and economical aspects [23, 24]. In addition, certain pathways into retirement have been previously related to economic differences [7, 25].

The first cluster contains *late labour market exits* after the age of 65. From the age of 65, most individuals in this cluster received some sort of pension but continued to work.

The second cluster is characterized by *early pension withdrawal*. Even though the majority in this cluster also continued working until later ages, pensions were withdrawn before the age of 64. This indicates that individuals in this cluster were more likely to decrease their paid work and complement their income with pension benefits from an early age. The age when this group stopped working completely was very flexible across the retirement phase.

The third cluster can be described as *standard retirement*. Most individuals in this group stopped working completely within a year. In terms of retirement age, transitions into full retirement were mainly completed at the age of 65. Earlier (partial) exits were common after the age of 63. This cluster contained slightly more women and lower educated individuals compared with the first two clusters, and disposable income was also somewhat lower.

The fourth cluster is described by *low income from work and early transitions into full retirement.* It did not differ much from the third cluster in terms of gender and educational constellation.

The fifth cluster describes *labour market exits via disability pensions*. This cluster differed from all other clusters by comprising the highest proportion of low-educated individuals and women, and by having the lowest disposable income.

### Correlates of sequences

**Figure 4.**
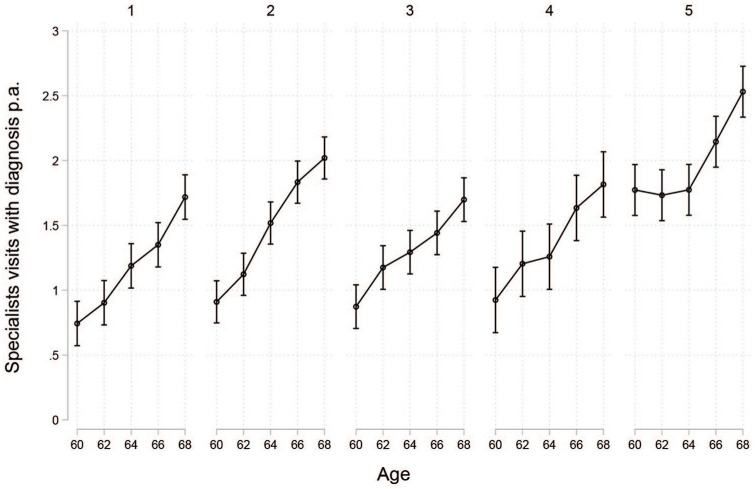
Number of specialist visits with diagnosis by cluster.

**Figure 5.**
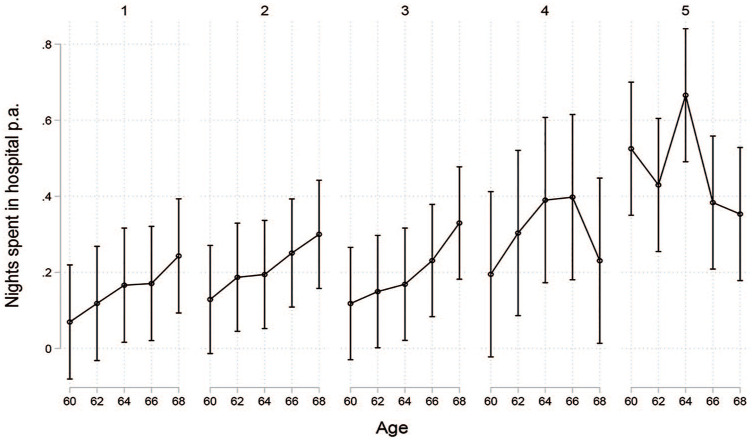
Number of nights in hospital by cluster.

Interestingly, the first two clusters, *late labour market exits* and *early pension withdrawal*, did not differ much in terms of social groups and socio-economic characteristics. Both included more highly educated individuals compared with the other clusters, had the highest disposable income and a larger proportion of men. Hence, these characteristics could not explain the different pathways. Looking at the health profiles shown in Figures 4 and 5, the distinction between these two groups becomes more apparent. The second cluster (*early pension withdrawal)* showed some possible health impairments early on in the retirement process. There was a steep early increase in visits to specialists before the age of 64. Furthermore, health at the beginning of the retirement phase seemed to be slightly worse. Hence, there seemed to be a health selection into the two different pathways: those with worse health or a decrease in health early in the retirement phase end up in the second cluster and withdraw some of their pensions early. However, this pattern was only visible in relation to visits to a specialist and was not reflected in the other measures of healthcare use.

Compared with the second cluster (*early pension withdrawal)*, the increase in number of specialist visits in the third cluster (*standard retirement*) seemed to be weaker, despite similar levels at the beginning of the retirement process. However, the third cluster also differed from the second in the constellation of social groups and was characterized by lower socio-economic status. Since low socio-economic status is related to lower use of specialist care [26], the lower income of the third cluster may partly explain the differing development of number of specialist visits over time in both pathways. Furthermore, looking at the number of nights spent in hospital, there was no indication of a more negative development of this healthcare measure in the second cluster.

In the fourth cluster (*low income from work and early transitions into full retirement*), disposable income was somewhat lower compared with the third cluster (*standard retirement*), however, there were no strong differences in the health profiles.

As expected, health at the beginning of the retirement phase seemed to be much worse in the fifth cluster (*labour market exits via disability pensions*) than in the other four clusters. Nights spent in hospital and number of visits to the doctor were much higher in this group. This cluster also showed some interesting development in terms of specialist use. Visits to a specialist at the age of 60 were already occurring as often as among those over the age 68 in the other clusters. There was no increase in specialist visits during the period of disability pension benefits, but there was a strong increase after shifting into old-age pension. This could be interpreted in light of the results from a large-scale Swedish study [27]. This study found out that 10% of those with disability pensions refrained from healthcare use due to financial reasons, a figure that was higher than in the general population. Looking at the income profiles of this cluster, their financial situation did not improve significantly over time. Hence, the difference in specialist visits during receipt of disability- and old-age pension is unlikely to be explained merely by disposable income.

Across all clusters, a clear and steady increase in specialist visits was observed, which may be ageing related rather than linked to labour force exit or pathway.

## Discussion

To our knowledge, this study is the first to describe different pathways into retirement for a full cohort in Sweden. To date, this has been done for Germany and Britain [28] and for the United States [29] for retirement cohorts around the late 1990s and early 2000s. Due to the different institutional contexts and cohorts, the here-examined more recent retirement cohorts might be expected to show a very different picture. In addition, while there was a greater focus on early retirement in these previous studies, our study points to a new phenomenon: late retirement pathways. The largest cluster in our study is one that has rarely been recognized in previous literature, as it is characterized by a more gradual exit with a combined pension and income from paid work.

Applying a clear income-based definition of retirement and identifying different clusters of pathways, the current study found some evidence that there may be health-related selection into different retirement pathways. Previous research on the effect of retirement on health has often focused on an abrupt transition from work into retirement, which is represented by the third cluster in the current study. Some studies also include a disability pathway, which is represented by our fifth cluster [5]. Our study found an often neglected heterogeneity of retirement transitions and that, in particular, a large proportion of employees in Sweden continues to work beyond age 65, at least to some degree, while receiving some sort of pension. Many arguments on the beneficial effects of retirement on health arise from the notion that a loss of work strain is related to better health. However, the current study made clear that considering receipt of the first pension as a measure of transition into retirement will lead to blurred results. The same would be true if retirement is understood as a complete exit from the labour market, as reduced work strain might already have occurred over several years of reduced labour market participation. In particular, with the current labour market reforms allowing for a stronger de-standardized retirement pathway, future research should take heterogeneity in timing and sequences into account. Besides the here-applied statistical methods of clustering and stronger theoretically deduced approaches [13], future research should at least differentiate between full- and partial exits.

An important limitation of our study was the assumption that healthcare use was strongly related to health. While it has been found that physical health predicts healthcare utilization [3], our study fell short of a direct conclusion in relation to the effect of retirement pathways on health.

Using register data has significant advantages with regard to the selectivityof the sample. Survey data on self-reported health are often impaired by selection bias. Analysing register data includes all individuals and provides highly accurate information on income sources and different measures of healthcare use. Healthcare use may have a socio-economic gradient that does not reflect actual healthcare need. Therefore, provision of education and income profiles in order to draw careful conclusions is crucial.

While the representation of the heterogeneity in the pathways to retirement was astonishing, the influence of different pathways on health was limited. In line with literature on the effect of retirement on health, we expected different healthcare use patterns across different pathways. However, we did not find distinct developments across different measures of healthcare use that could be related to different pathways.

## Supplemental Material

sj-pdf-1-sjp-10.1177_1403494821998901 – Supplemental material for Development of healthcare use across contemporary retirement pathways: results from a register based cohort study Click here for additional data file. Supplemental material, sj-pdf-1-sjp-10.1177_1403494821998901 for Development of healthcare use across contemporary retirement pathways: results from a register based cohort study by Stefanie König, Susanne Kelfve, Andreas Motel-Klingebiel and Martin Wetzel in Scandinavian Journal of Public Health

## References

[1] van den Berg TIJ Elders LAM Burdorf A . Influence of health and work on early retirement. J Occup Environ Med 2010;52:576–83. 10.1097/JOM.0b013e3181de8133 20523241

[2] van der Heide I van Rijn RM Robroek SJ , et al. Is retirement good for your health? A systematic review of longitudinal studies. BMC Public Health 2013;13:1180. 10.1186/1471-2458-13-1180 PMC4029767 24330730

[3] Fylkesnes K . Determinants of health care utilization—visits and referrals. Scand J Soc Med 1993;21:40–50. 8469943 10.1177/140349489302100107

[4] Genbäck M Ng N Stanghellini E , et al. Predictors of decline in self-reported health: addressing non-ignorable dropout in longitudinal studies of aging. Eur J Ageing 2018;15:211–20. 10.1007/s10433-017-0448-x PMC5971030 29867305

[5] Halleröd B Örestig J Stattin M . Leaving the labour market: the impact of exit routes from employment to retirement on health and wellbeing in old age. Eur J Ageing 2012;10:25–35. 28804280 10.1007/s10433-012-0250-8 PMC5549229

[6] Sewdas R van der Beek AJ de Wind A , et al. Determinants of working until retirement compared to a transition to early retirement among older workers with and without chronic diseases: results from a Dutch prospective cohort study. Scand J Public Health 2017;46:400–8. 10.1177/1403494817735223 PMC5946665 29059016

[7] Polvinen A Laaksonen M Gould R , et al. Socioeconomic inequalities in cause-specific mortality after disability retirement due to different diseases. Scand J Public Health 2015;43:159–68. 10.1177/1403494814562597 25504585

[8] Calvo E Sarkisian N Tamborini CR . Causal effects of retirement timing on subjective physical and emotional health. J Gerontol B Psychol Sci Soc Sci 2013;68:73–84. 23149431 10.1093/geronb/gbs097

[9] Kelfve S Lennartsson C Agahi N , et al. Do postal health surveys capture morbidity and mortality in respondents aged 65 years and older? A register-based validation study. Scand J Public Health 2015;43:348–55. 10.1177/1403494815575340 25754866

[10] Denton FT Spencer BG . What is retirement ? A review and assessment of alternative concepts and measures. Can J Aging 2009;28:63–76. 19860967 10.1017/S0714980809090047

[11] König S Lindquist GS . Sweden: steeply rising older workers’ employment rates in a late-exit country. In: Hofäcker D Hess M König S (eds) Delaying Retirement. London: Palgrave Macmillan, 2016, pp. 315–35.

[12] Laun T Wallenius J . A life cycle model of health and retirement: the case of Swedish pension reform. J Public Econ 2015;127:127–36.

[13] Schmälzle M Wetzel M Huxhold O . Pathways to retirement: are they related to patterns of short- and long-term subjective well-being? Soc Sci Res 2019;77:214–29. 10.1016/j.ssresearch.2018.10.006 30466876

[14] Madero-Cabib I Fasang AE . Gendered work–family life courses and financial well-being in retirement. Adv Life Course Res 2016;27:43–60.

[15] Riekhoff A-J . Extended working lives and late-career destabilisation: a longitudinal study of Finnish register data. Adv Life Course Res 2018;35:114–25.

[16] Wahrendorf M Zaninotto P Hoven H , et al. Late life employment histories and their association with work and family formation during adulthood: a sequence analysis based on ELSA. J Gerontol Ser B Psychol Sci Soc Sci 2018;73:1263–77 10.1093/geronb/gbx066 PMC6146763 28575487

[17] Fasang AE . Retirement: institutional pathways and individual trajectories in Britain and Germany. Sociol Res Online 2010;15:1–16.

[18] Riekhoff A-J Järnefelt N . Gender differences in retirement in a welfare state with high female labour market participation and competing exit pathways. Eur Sociol Rev 2017;33:791–807.

[19] Ludvigsson JF Andersson E Ekbom A , et al. External review and validation of the Swedish national inpatient register. BMC Public Health 2011;11:450. 21658213 10.1186/1471-2458-11-450 PMC3142234

[20] Jönsson L Palme M Svensson I . Disability insurance, population health and employment in Sweden, www.nber.org/papers/w17054 (2011, accessed October 13 2017).

[21] Brzinsky-Fay C Kohler U Luniak M. Sequence Analysis with Stata. Stata J Promot Commun Stat Stata 2006;6:435–60.

[22] Studer M Ritschard G. A comparative review of sequence dissimilarity measures. LIVES Working Papers. Epub ahead of print 2014. DOI: 10.12682/LIVES.2296-1658.2014.33.

[23] Cameron KA Song J Manheim LM , et al. Gender disparities in health and healthcare use among older adults. J Womens Health 2010;19:1643–50. 10.1089/jwh.2009.1701 PMC2965695 20695815

[24] Veugelers PJ Yip AM . Socioeconomic disparities in health care use: does universal coverage reduce inequalities in health? J Epidemiol Community Health 2003;57: 424–8. 10.1136/jech.57.6.424 PMC1732477 12775787

[25] Leinonen T Martikainen P Lahelma E . Interrelationships between education, occupational social class, and income as determinants of disability retirement. Scand J Public Health 2012;40:157–66. 10.1177/1403494811435492 22312029

[26] Dunlop S Coyte PC McIsaac W . Socio-economic status and the utilisation of physicians’ services: results from the Canadian National Population Health Survey. Soc Sci Med 2000;51:123–33. 10.1016/s0277-9536(99)00424-4 10817475

[27] Molarius A Simonsson B Lindén-Boström M , et al. Social inequalities in self-reported refraining from health care due to financial reasons in Sweden: health care on equal terms? BMC Health Serv Res 2014;14:605. 25468266 10.1186/s12913-014-0605-2 PMC4254004

[28] Fasang AE . Retirement: institutional pathways and individual trajectories in Britain and Germany. Sociol Res Online 2010;15:1–16.

[29] Calvo E Madero-Cabib I Staudinger UM . Retirement sequences of older Americans: moderately destandardized and highly stratified across gender, class, and race. Gerontologist; 2018;58:1166–76. 10.1093/geront/gnx052 PMC7594171 28586409

