# Supplementary material for: Development of healthcare use across contemporary retirement pathways: results from a register based cohort study
Source: Scand J Public Health. 2021 Mar 19;50(4):440–7. doi: 10.1177/1403494821998901 (PMC9152596; doi:10.1177/1403494821998901)

Methodological appendix

Many distance measures have been applied in sequence analysis to identify the similarity between the sequences (Studer & Ritschard, 2015). Based on the focus of the analysis in terms of focus on timing, sequencing or duration, they show differences in precession of the clustering. In the current paper we aim to differentiate sequences mostly based on the timing which can be understood as the age at which different states (and therewith different transition between states) occur. In particular, we attempt to classify individuals who start to receive pension payments (partially or fully) at different ages into distinct classes. Although these different focusses of sequences analysis are interrelated, the current study focusses less on the duration understood as the length in on particular state and less sequencing understood as the ordering of different states since the huge majority of the sample population follows a similar succession of states starting with employment based income after which the income sources become more and more pension based ending with fully pension-based income. A simulation study has shown that the often-applied (classical) Optimal Matching is well-suited for this purpose (Studer & Ritschard, 2015).

In the Optimal Matching approach, distance between two sequences is calculated based on substitution and indel costs. Substitution costs try to depict how similar two states are. The substitution of two similar states should be associated with less costs than of two states which are very distinct. For our approach, we found no conceptual assumptions for identifying different costs for different status changes. For instance, a substitution in a sequence of income-based states from "fully salary-based" to "fully pension-based income" should not differentiate stronger these sequences as a substitution between the states "receiving more than 50 percent of the income from salary" to "receiving less than 50 percent of the income from salary". This was very similar to what we found when we applied a data-driven approach using a transition probabilities matrix. The assumption behind this probability matrices approach is that more often occurring transitions between two statuses are more similar and hence should have lower substitution cost. We found that the different states have very similar transition probabilities which translated to substitution cost between all states of around 2. We finally decided to apply the substitution costs from the probability matrix. Distances between sequences in the

Optimal Matching approach depend also on the indel costs which represent the costs of inserting or deleting a state to harmonize two sequences.

Based on these definitions, we followed the state of the art in OM and estimated the Levenshtein distance for each individual with each other individuals. To identify distinct classes, we used Ward-clustering which identifies gradually similar classes. The results are shown in the dentogram of Figure Appendix F1. The decision on the number of classes is based on the conflict of targets to reduce complexity while differentiating between different patterns. Based on the dentogram, we decided to opt for a 5-class solution. A more detailed differentiation would have led directly to a 7-class solution (differentiating class 2 and class 4) which would have reduced the size of the smallest class to below 5 % of the sample population. Finally, the applied method led for the interpretation to consistent pattern between the classes and a generally plausible result which are an important characteristic for the success of the sequence analysis.

Figure Appendix F1) Dentogram for the classification of Levenshtein-distances between sequences

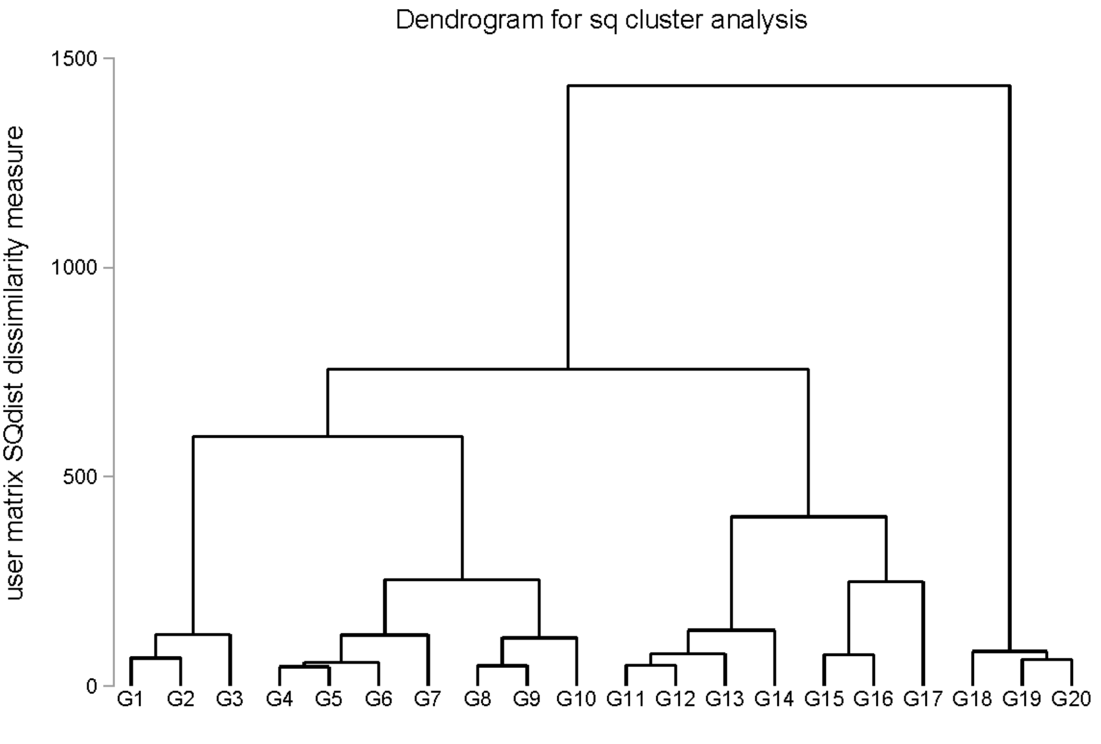

Supplement: sj-pdf-1-sjp-10.1177_1403494821998901 – Supplemental material for Development of healthcare use across contemporary retirement pathways: results from a register based cohort study [file sj-pdf-1-sjp-10.1177_1403494821998901.pdf]
